# Disseminated tuberculosis with lymphatic, splenic and scrotal abscesses: a case report

**DOI:** 10.4076/1757-1626-2-6995

**Published:** 2009-08-05

**Authors:** Ergin Ayaslioglu, Halil Basar, Nihal Duruyurek, Fusun Kalpaklioglu, Sedef Gocmen, Arzu Erturk, Sinasi Yilmaz

**Affiliations:** 1 Department of Infectious Diseases and Clinical Microbiology, Kirikkale University School of Medicine71100, KirikkaleTurkey; 2 Department of Urology, Kirikkale University School of MedicineKirikkaleTurkey; 3 Department of Pulmonary Diseases, Kirikkale University School of Medicine71100, KirikkaleTurkey; 4 Department of Clinical Microbiology, Kirikkale University School of Medicine71100, KirikkaleTurkey; 5 Department of Respiratory Medicine, Ataturk Chest Disease and Surgery Center06280, AnkaraTurkey; 6 Department of Pathology, Kirikkale Yuksek Ihtisas Hospital71400, KirikkaleTurkey

## Abstract

**Introduction:**

Disseminated tuberculosis can involve several organs and clinically present with a potpourri of signs and symptoms. Early diagnosis and timely initiation of proper treatment are of great importance in preventing the later complications of the disease.

**Case presentation:**

We report a case with disseminated tuberculosis who exhibited a wide spectrum of extrapulmonary involvement. The present case had lung and lymph node tuberculosis with abscess formation and remained undiagnosed for two years. Thereafter, multiple splenic abscesses developed that necessitated splenectomy, and at the final stage, he presented with scrotal abscesses.

**Conclusion:**

This paper highlights the diverse clinical appearances of disseminated tuberculosis and the significant importance of early diagnosis and treatment.

## Introduction

Disseminated tuberculosis (TB) is defined as having two or more noncontiguous sites resulting from lymphohematogenous dissemination of *Mycobacterium tuberculosis*. It has become more common in most developed countries due to the advent of human immunodeficiency virus (HIV) infection. However, the rate of late and complicated cases is high in developing countries due to the poor health conditions. Because the clinical manifestations are quite nonspecific, it can mimic several other disorders, and diagnosis is often difficult. A high index of suspicion and familiarity with its diverse features allow early recognition and treatment of the disease [1-2].

## Case presentation

**Figure 1.**
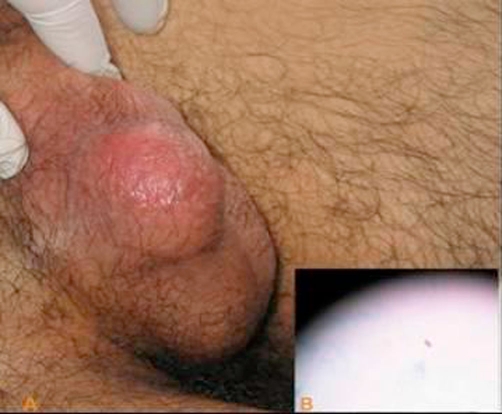
**(A)** Tuberculous epididymitis clinically presents as a scrotal abscess. **(B)** Positive acid fast staining of scrotal abscess.

A 25-year-old male from a central Anatolian village was admitted to the Urology Department with pain and swelling in his scrotum for five days (Figure 1A).

Three years before, he had complaints of fatigue, weakness, and weight loss (approximately 30 k), as well as persistent sweating and cough. However, he did not admit to any hospital until a right axillary painful lymphadenopathy (LAP), of approximately 4 × 5 cm, had appeared. Nonspecific antibiotic treatment was begun immediately after drainage (performed twice) and the LAP regressed.

**Figure 2.**
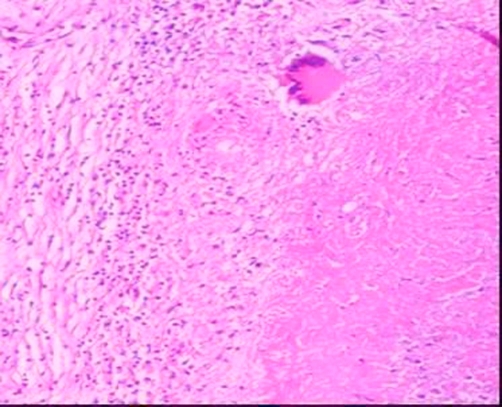
Splenic granuloma showing caseous necrosis, giant cells and lymphocytes at the edge (Original magnification ×400).

He was free of symptoms for two and a half years. Thereafter, he began to complain of abdominal pain, which over time became severe. Two months ago, he admitted to the emergency department with severe abdominal pain, nausea and vomiting, and abdominal distention, and was operated on an emergency basis. During the operation, the spleen was found to be enlarged with multiple abscesses of varying size, one of which ruptured. Mesenteric material also showed nodular lesions. Splenectomy was performed. Histopathological examination obtained two weeks later revealed a granulomatous reaction with caseous necrosis (Figure 2).

A short time following discharge from the hospital, a painful swelling appeared in his right scrotum and he was hospitalized with scrotal abscess in the urology clinic. His temperature on admission was 37.2°C and did not increase during the follow-up. The patient was slim in appearance without sweating, cough, or sputum. His physical examination revealed right axillary LAP of 1 cm and multiple minimal inguinal LAPs. The incision site of the previous abdominal operation was still draining. His genitourinary examination showed a painful, soft, fluctuant and erythematous mass of 4 × 5 cm, suggestive of a scrotal abscess. Complete blood count revealed: white blood cells (12,500/mm^3^), hemoglobin (12.6 g/dL) and platelets (901/ mm^3^). Erythrocyte sedimentation rate and C-reactive protein showed an increase at 75 mm/hr and 66.8 mg/dL, respectively. Urine analyses revealed microscopic hematuria and pyuria.

There were no associated systemic disorders, and HIV infection was not detected. Purified protein derivative of tuberculin test (PPD) was anergic. Chest radiography showed occluded right costophrenic sinus with minimal pleural thickening in the right apex. Computed tomography scan of the lungs revealed 2-3 subpleural nodules (largest: 1.5 × 2 cm) in the right upper lobe with apicoposterior localization. These findings were not definitive for active disease, but indicated past disease. In ultrasonographic examination, testes were bilaterally normal, although the right epididymis was enlarged in size and in vascularity, suggestive of epididymitis. Minimal ascites was also detected on abdominal examination.

After drainage of the scrotal abscess (approximately 10-20 cc), specific and non-specific cultures were taken. Direct staining of the drainage material showed acid fast bacilli (AFB) (Figure 1B). Conventional culture method using Lowenstein-Jensen medium also yielded the mycobacterium several days later. A treatment with standard 4-drug regimen of DOT (directly observed treatment) was initiated as isoniazid 300 mg, rifampin 600 mg, ethambutol 1500 mg and pyrazinamide 2000 mg for two months, followed by a 2-drug regimen for 10 months. One month later, the scrotal abscess had gradually subsided and the patient was discharged. After 12 months, he was completely healthy with bilaterally normal testes and a weight gain of approximately 20 kg.

## Discussion

Tuberculosis is a broad spectrum disease that may involve pulmonary and extrapulmonary locations. Lymph nodes have been generally reported as the most common nonpleural site [3,4]. This form is generally multifocal, with a peak onset during childhood and young adulthood [1,5]. Although it is a very common extrapulmonary presentation, lymph TB of the present case was misdiagnosed and treated as a nonspecific abscess with drainage and antibiotics. Two years later, the patient presented with splenic and scrotal abscesses. Therefore, a high index of suspicion for TB is necessary for the diagnosis.

Genitourinary TB is the second most common extrapulmonary form of TB after peripheral LAP. Up to 20% of the patients with pulmonary TB have genitourinary lesions, particularly in the kidneys [6,7]. *M. tuberculosis* generally reaches the genitourinary organs, by hematogenous route, disseminating from primary pulmonary TB. However, urologic spread of renal foci may infect genital organs: the prostate, seminal vesicles, epididymis and testes [8]. Persistent sterile pyuria and hematuria are the most classical findings that can be seen in urogenital TB. In the case of epididymal TB, pain, swelling, heaviness or mass lesion in the scrotum are the common presenting complaints [6,7]. Our case presented with a painful scrotal mass that was initially drained as a nonspecific abscess. Epididymal TB was diagnosed after acid fast resistant bacilli was shown in direct examination of the drainage material.

Splenic TB, especially an abscess formation, is an unusual manifestation and usually occurs in patients with concurrent HIV infection [5,9]. However, it may be found in severe, disseminated disease in non-HIV patients, as in the presented case. Clinical findings are nonspecific and TB presents as unexplained fever, abdominal pain and splenomegaly. Abdominal pain and distention, together with nausea and vomiting, were the leading symptoms in the present case. Splenomegaly was also present. The treatment is generally splenectomy in the event of multiple splenic abscess and splenic rupture [9,10]. AntiTB drugs must be used as complementary treatment especially when associated with mesenteric involvement.

Patients with suspected TB should have appropriate specimens sent for acid fast bacilli staining, mycobacterial culture, and histopathological examination [8,9]. Although microbiologic staining has a low sensitivity, the diagnosis of TB in this case was made following direct examination of the scrotal material with acid fast bacilli staining, as well as the positive culture, which is a slow process. Moreover, pathological examination of his splenectomy material, which was consistent with caseating granulomatous inflammation, confirmed the TB diagnosis.

## Conclusion

Tuberculosis remains one of the most fatal diseases in the world, making early diagnosis and appropriate treatment of critical importance. Our patient was unluckily not diagnosed for three years because of insufficient medical aid until the disseminated disease had manifested with multi-organ involvement. After initiation of antiTB therapy, he showed marked recovery with a 12-month course of DOT regimen, which enhances the patient compliance. We believe that this case with its broad clinical presentation emphasizes the significant importance of early diagnosis and treatment in TB.

## Abbreviations

AFB: acid fast bacilli
DOT: directly observed treatment
HIV: human immunodeficiency virus
LAP: lymphadenopathy
PPD: purified protein derivative of tuberculin test
TB: tuberculosis
